# Vitamin A Deficiency and the Lung

**DOI:** 10.3390/nu10091132

**Published:** 2018-08-21

**Authors:** Joaquín Timoneda, Lucía Rodríguez-Fernández, Rosa Zaragozá, M. Pilar Marín, M. Teresa Cabezuelo, Luis Torres, Juan R. Viña, Teresa Barber

**Affiliations:** 1 Department of Biochemistry and Molecular Biology, Faculty of Pharmacy, University of Valencia, Avgda V. Andrés Estellés s/n, 46100 Burjassot, Spain; joaquin.timoneda@uv.es; 2 Department of Biochemistry and Molecular Biology, Faculty of Medicine-IIS INCLIVA, University of Valencia, Avda. Blasco Ibañez, 15, 46010 Valencia, Spain; roferlu@uv.es (L.R.-F.); luis.torres@uv.es (L.T.); juan.r.vina@uv.es (J.R.V.); 3 Department of Human Anatomy and Embriology, Faculty of Medicine-IIS INCLIVA, University of Valencia, Avda. Blasco Ibañez, 15, 46010 Valencia, Spain; rosa.zaragoza@uv.es; 4 Microscopy Unit IIS La Fe Valencia, Avda Campanar, 21, 46009 Valencia, Spain; marin_marmue@gva.es; 5 Department of Physiology, Faculty of Medicine, University of Valencia, Avda. Blasco Ibañez, 15, 46010 Valencia, Spain; tecabar@alumni.uv.es; 6 Centro Salud Valencia-Ingeniero J. Benlloch, C/Joaquin Benlloch, 27, 46006 Valencia, Spain; 7 Hospital Universitario Doctor Pesset, Gaspar Aguilar, 90, 46017 Valencia, Spain

**Keywords:** Vitamin A deficiency, retinol, retinoic acid, lung, pulmonary disease, extracellular matrix, E-cadherin, N-cadherin, collagen, epithelial–mesenchymal transition

## Abstract

Vitamin A (all-*trans*-retinol) is a fat-soluble micronutrient which together with its natural derivatives and synthetic analogues constitutes the group of retinoids. They are involved in a wide range of physiological processes such as embryonic development, vision, immunity and cellular differentiation and proliferation. Retinoic acid (RA) is the main active form of vitamin A and multiple genes respond to RA signalling through transcriptional and non-transcriptional mechanisms. Vitamin A deficiency (VAD) is a remarkable public health problem. An adequate vitamin A intake is required in early lung development, alveolar formation, tissue maintenance and regeneration. In fact, chronic VAD has been associated with histopathological changes in the pulmonary epithelial lining that disrupt the normal lung physiology predisposing to severe tissue dysfunction and respiratory diseases. In addition, there are important alterations of the structure and composition of extracellular matrix with thickening of the alveolar basement membrane and ectopic deposition of collagen I. In this review, we show our recent findings on the modification of cell-junction proteins in VAD lungs, summarize up-to-date information related to the effects of chronic VAD in the impairment of lung physiology and pulmonary disease which represent a major global health problem and provide an overview of possible pathways involved.

## 1. Introduction

Vitamin A (all-*trans*-retinol) is a fat-soluble micronutrient which, together with its natural derivatives and synthetic analogues that exhibit its biological activity, constitutes the group of retinoids [1]. It is converted by two successive oxidative reactions into its main biologically active derivatives, retinaldehyde and retinoic acid (RA), which can exist as all-*trans* or several *cis* isomers. Vitamin A is the most multifunctional vitamin in the human body, as it is involved in several essential physiological processes from embryogenesis to adulthood. Most of these functions are not carried out by retinol itself but by its active metabolites. They have a number of already established functions including vision, immunity, cell differentiation, embryological development, cellular differentiation and proliferation and antioxidant function [2,3,4,5,6,7]. The importance of vitamin A in regulating growth through cell proliferation and differentiation was recognized early in the 20th century [8,9]. In the last decade, new biological functions related to insulin resistance, lipid metabolism, energy balance and redox signalling have been described [10,11].

RA exerts its broad range of biological effects mainly by controlling gene expression. RA binds to and activates two classes of nuclear ligand-dependent transcriptional regulators that belong to the superfamily of nuclear specific receptors and that comprise two subfamilies, RARs (RA receptors) and RXRs (retinoid X receptors). Both classes of receptors have three subtypes (α, β and γ) and each of them has different isoforms. RARs require heterodimerization with RXRs for DNA binding and subsequent function and are activated by all-*trans*-retinoic acid (atRA), the predominant isomer of RA in vivo and by 9-*cis*-retinoic acid (9-*cis*-RA), whereas RXRs are only activated by 9-*cis*-RA. RAR-RXR heterodimers bind to specific DNA regions, termed RA response elements (RAREs) in the promoter region of target genes resulting in the control of transcription [12]. RA can also bind other nuclear receptors, such as the peroxisome proliferation-activated receptor β/δ (PPAR β/δ), which participate in the regulation of energy homeostasis and insulin response. Over 500 genes have been reported to be responsive to either all-*trans*- or 9-*cis*-RA. Moreover, RA also has extranuclear, nontranscriptional direct effects, which influences the expression of RA target genes via phosphorylation processes. In addition, different transcriptional effects for retinol and retinal have also been described [10,13,14].

Although a considerable effort in the control of vitamin A deficiency (VAD) has been made in the last years, VAD is still a major public health problem in the world and has important implications for the global health policy. According to the World Health Organization [15], VAD constitutes, with protein malnutrition, the most common nutritional disorder in the world. It is estimated that 250 million preschool-aged children in developing countries are VAD and 5 million are clinically affected by this deficiency. A value below a cut-off of 0.70 µmol/L of serum retinol concentrations represents biochemical VAD; in these persons, the tissue concentrations of this vitamin are low enough to result in increased risk of adverse health effects. Serum retinol concentrations lower than 0.35 μmol/L are indicative of severe deficiency and are associated with marked increases in the risk of clinical manifestations, including xerophthalmia accompanied by nyctalopia, hyperkeratosis, increased susceptibility to severe infection and disturbances in cell differentiation, organ development, growth and reproduction [16,17,18]. It is important to point out that over 20% of the population in the developed world does not reach two-thirds of the recommended intake and has plasma and liver concentrations of vitamin A lower than those accepted as normal as a result of modern societal habits. Therefore, in these countries it is important to consider the subclinical deficiency [19,20,21]. VAD, even the asymptomatic subclinical form, increases morbidity and mortality from several infections and also increases the incidence and morbidity of respiratory tract illnesses [17,22,23,24,25].

Vitamin A is involved in the proliferation and maintenance of epithelial cells, including those of the respiratory tract. It is a major factor regulating differentiation and maturation of the lung, and maternal VAD during pregnancy could have lasting adverse effects on the lung health of the offspring [26]. Early retinoid deprivation of pregnant animals results in abnormalities such as lung agenesis and later deprivation results in defective alveologenesis [27]. Vitamin A is also required for the maintenance of alveolar architecture and tissue regeneration after the alveoli have been formed. Available evidence showing that nutritional VAD induces negative histologic changes within the respiratory tract indicates that retinoids continue to play an important role in the adulthood lung [23,28,29].

VAD has been associated with histopathological changes in the pulmonary epithelial lining and in lung parenchyma which leads to disrupt the normal lung physiology and predisposes to severe tissue dysfunction and respiratory diseases. These alterations are associated with changes in the extracellular matrix (ECM) and basement membrane (BM) protein content and distribution [30,31,32]. ECM provides tissues with structural strength and flexibility and cells with attaching support. In addition, ECM also accomplishes important signalling functions. It is formed by a complex array of highly cross-linked macromolecules, such as collagens, laminins, fibrilins, elastins, fibronectins and several proteoglycans. In the lung, ECM maintains tensile strength, elasticity and dictates the specialized function of multiple cell lineages. BMs, which are laminar structures of highly specialized ECM, are associated with the airway and alveolar epithelium, endothelium, nerve cells and visceral pleura, bronchial and vascular smooth muscle cells. In the alveoli, they are also part of the air-blood barrier and thence traversed during the gas-exchange process. We have reported that collagens I and IV show an increase in the lungs of VAD rats and, in parallel, the alveolar BM doubles in thickness and appears to have an ectopic deposition of collagen I fibrils inside. These morphological alterations might be a contributory factor to the development or progression of lung disease [32,33,34].

In this review, we report our recent findings on the modification of cell-junction proteins in VAD lungs and summarize the information available on the effects of chronic VAD in the impairment of lung physiology and pulmonary disease, which represent a major global health problem [35] and provide an overview of the possible pathways involved.

## 2. Vitamin A Metabolism and Retinoid Signalling

### 2.1. Vitamin A Bioavailability and Requirements

Vitamin A cannot be synthesized by vertebrates, including humans but instead has to be obtained as a micronutrient from the diet. There are a large number of sources of dietary vitamin A. Vitamin A is present in foods either as preformed vitamin A, mainly retinyl esters (RE) but also all-*trans*-retinol, which can be obtained from animal sources such as milk, eggs, liver and food products fortified with vitamin A, or as provitamin A, which refers to some carotenoids, mainly β-carotene, which are partly converted to vitamin A in the intestinal mucosa, as well as in other peripheral non-digestive tissues (e.g., adipocytes, macrophages) [36,37] and abounds in darkly coloured fruits and vegetables as green leaves, carrots, ripe mangos and other orange-yellow vegetables and fruits. Although retinyl esters and all-*trans*-retinol are the most abundant preformed retinoids in the diet, some RA is also present but normally it represents under 0.01% of the total preformed retinoid content in a common diet [38].

The requirements for vitamin A are based on the assurance of adequate liver stores of vitamin A (>20 µg/g liver) and are actually expressed in retinol activity equivalents (RAE). One µg of RAE is defined as the biological activity associated with 1 µg of all-*trans*-retinol and, based on its estimated efficacy of absorption and conversion into vitamin A, it is equivalent to 12 µg of β-carotene and to 24 µg of α-carotene or β-cryptoxanthin (other carotenoids found in food, such as lycopene, lutein and zeaxanthin, are not metabolic precursors for vitamin A). This change of bioconversion means that a larger amount of provitamin A carotenoids and, therefore, higher amount of carotene-rich fruit and vegetables, is needed to meet vitamin A requirements. Therefore, in populations from low-income countries, to depend solely on vegetable intakes for vitamin A sources increases the risk of VAD. In addition, carotenoids could be beneficial when ingested in physiological amounts but may have side effects when administered at high doses or under a highly oxidative status. It has been estimated that individuals in developing nations receive 70%–90% of their retinoid from provitamin A carotenoids, whereas individuals in industrialized nations consume up to 75% of their total dietary retinoid as preformed vitamin A, including the intake of vitamin A added to dietetic products. Vitamin A levels could also be stated as international units (IU). One IU is equivalent to 0.3 µg of all-*trans*-retinol, or 0.3 µg of RAE. The recommended dietary allowances (RDAs) for children, men and women are 300–600, 900 and 700 µg of RAE/day, respectively. During pregnancy, the RDA is 750 mg of RAE/day and increases to 1300 mg of RAE/day during lactation. There is no RDA for β-carotene or other provitamin A carotenoids. Finally, it is important to consider that a number of factors can affect the vitamin A absorption and availability and thus its requirements, including the presence and severity of infection and parasites, intestinal or liver disease (such as biliary atresia, cholangitis, viral hepatitis, alcoholic liver disease and non-alcoholic fatty liver disease), iron and zinc status, stress, fat intake, xenobiotics, protein energy malnutrition, alcohol consumption and the food matrix and food processing. Both insufficient dietary retinoid intake (hypovitaminosis A or VAD) and excessive retinoid consumption resulting in vitamin A concentrations above the physiological range (hypervitaminosis A or vitamin A-toxicity) cause adverse effects to human health, which are paradoxically similar in both situations [24,38,39,40,41,42,43,44,45].

### 2.2. Vitamin A Uptake, Transport and Metabolism

Retinol, RE and carotenoids absorption depends both on common lipid absorption and on specific enzymes, binding proteins and transporters. Dietary retinol and RE are efficiently absorbed by duodenal mucosal cells after solubilizing into micelles in the lumen of the intestine. Retinol is directly taken up by enterocytes whereas RE are unable to be absorbed intact by the intestinal mucosa and are hydrolysed to retinol by extracellular RE hydrolases. After absorption by enterocytes, by passive diffusion or by a process that may involve a membrane transporter, they are re-esterified in the gut by lecithin: retinol acyltransferases to RE and delivered through circulating chylomicrons mainly to the liver. Unesterified retinol is also absorbed into the portal circulation and its efflux from the basolateral cell membrane is facilitated by the lipid transporter ABCA1. On the other hand, β-carotene is less well absorbed than the preformed RE. β-carotene is taken up in enterocytes by membrane-bound transporters, probably involving scavenger receptor class B type I, SR-BI and then it must be cleaved and converted to retinol, which is esterified as RE and incorporated, together with a fraction of the absorbed carotenoids, into chylomicrons. The major pathway implicated in the rupture of β-carotene is the central cleavage catalysed by a cytosolic enzyme, β-carotene 15,15′-oxygenase 1; this enzyme cleaves β-carotene at its central double bond (15,15′) to yield retinal, which is then reduced to retinol by a retinal reductase. Post-prandial chylomicrons are secreted into the lymphatic system and from here to the general circulation through the thoracic duct. Before clearance by peripheral tissues, RE must first be hydrolysed to retinol by lipoprotein lipase (LPL). It is stablished that chylomicron RE is taken up mainly by the liver, where chylomicron remnants are cleared from the circulation, while a minor proportion is delivered to peripheral tissues during chylomicron remodelling by LPL. In healthy individuals, approximately 90% of vitamin A in the body is stored in the liver as RE. While parenchymal cells (or hepatocytes) are important for retinol uptake and mobilization, they account only for 10%–20% of the total retinoid found in the liver. The remaining 80%–90% of hepatic retinoid is found in the non-parenchymal hepatic stellate cells (also known as Ito cells, lipocytes or fat-storing cells), located in the space of Disse between the sinusoidal endothelial cells and hepatic epithelial cells. When required, vitamin A reserves are mobilized, hydrolysed back to retinol and transported in blood by the plasma carrier retinol-binding protein (RBP, also known as RBP4) to meet tissue needs, representing the predominant retinoid specie in the fasting circulation. The hepatocyte is the major cellular site within the liver that is able to synthesize and secrete retinol bound to RBP4. The retinol-RBP4 complex (holo-RBP) secreted from the hepatocyte into the circulation associates in plasma with another protein, transthyretin (transporter of thyroxin and retinol, TTR) (1:1), thus stabilizing the retinol-RBP4 complex, reducing renal filtration of the low molecular weight RBP4 and allowing for RBP4 to be recycled after retinol is taken into cells. The existence of stellate cells in other organs, such as pancreas, intestine, eyes, lungs and kidneys, suggests that they may be adapted to store vitamin A and also express and secrete RBP. Finally, different forms of retinoid have been found in the circulation in addition to retinol-RBP4 and RE (transported in chylomicrons and chylomicron remnants); these forms include RE transported from the liver in very low-density lipoprotein, VLDL and low-density lipoprotein, LDL, RA bound to albumin and retinol and RA transported in the form of water-soluble retinyl and retinoyl-β-glucuronides. However, in fasting conditions, retinol-RBP4 is the preponderant form comprising >95% of retinoids, whereas, after a retinoid-rich meal, chylomicron retinyl ester concentrations exceed those of retinol-RBP4. Regarding retinol transport into the cell, although it enters by free diffusion due to its hydrophobic nature, there is also evidence that supports retinol uptake by a protein located on the cell surface membrane. STRA6 (stimulated by RA 6) has been suggested as one possible receptor located in the plasma membrane for all-*trans*-retinol-RBP (holo-RB4) in many tissues, although its functions are complex and controversial. While STRA6 is a retinol transporter, it is not required for maintaining proper vitamin A levels in most tissues, neither during embryonic development nor in the adulthood. However, STRA6 mediated vitamin A uptake is a regulated process mandatory for ocular vitamin A uptake when RBP4 constitutes the only transport mode in VAD. Moreover, in addition to mediating vitamin A transport, STRA6 is also a surface signalling receptor when activated by holo-RBP4 it induces Janus kinases (JAK) 2 phosphorylation, triggering JAK2/signal transducers and activators of transcription (STAT) 3/5, a cascade that culminates in induction of STAT target genes within the nucleus. Thus, holo-RBP4 functions as a classical cytokine to activate STRA6/JAK2/STAT3/5 pathway. Within cells, retinol is reversibly oxidised to retinal by enzymes of the alcohol dehydrogenase, ADH and short-chain dehydrogenase/reductase families, SDR/RDH. RA is formed by irreversible oxidation of retinal catalysed by cytosolic aldehyde dehydrogenase 1 isoenzymes, ALDH1, alias RALDH. RA is further oxidized for excretion by mono-oxygenases of the cytochrome P450 (CYP) family. There are cellular binding proteins for retinol and retinal (CRBP) and RA (CRABP) which facilitate their metabolism and action. CRABP1 and CRABP2 seem to be involved in RA degradation and RA transcriptional activity, respectively. The metabolites from CYP degradation of retinol and RA are conjugated in the liver mainly with glucuronic acid and are excreted in bile and urine [38,46,47,48,49,50,51,52,53,54,55]. The treatment of human fibroblast with different tocopherols in the presence of retinol cause an increase in CRABP-2 mRNA and protein [56,57].

### 2.3. Retinoid Signalling

Retinoids and its natural derivatives retinol, retinal and RA, are involved in many important physiological functions, such as the vision, immunity, reproduction, embryonic development, cellular differentiation, tissue architecture maintenance, antioxidant function, redox signalling or energy balance [2,3,4,5,6,7,10,11,58].

**Figure 1:**
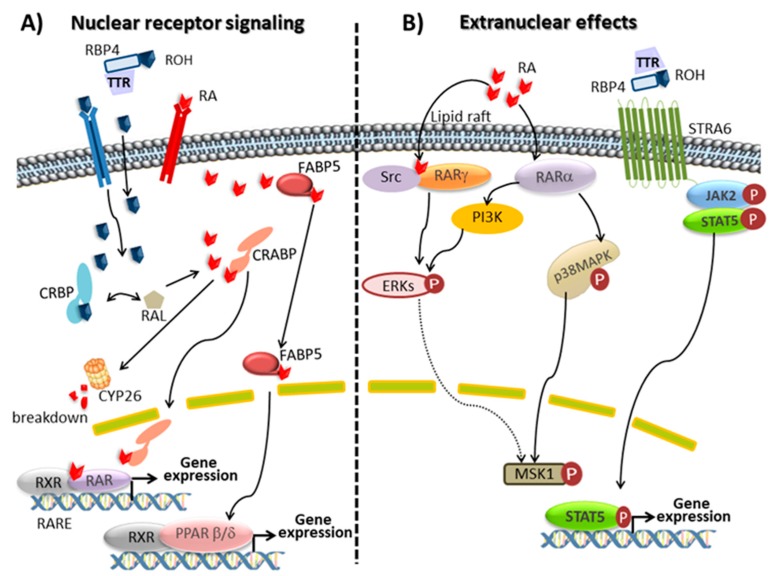
Intracellular signalling pathways of vitamin A. (**A**) Nuclear receptor signalling pathway. In blood, hydrophobic retinol (ROH) is bound by retinol-binding protein (RBP4) and transthyretin (TTR) and retinoic acid (RA) is bound to albumin. They enter the cell through membrane diffusion or ROH via the membrane transporter stimulated by RA (STRA6). Inside the cell, ROH is found in cytosol bound to cellular retinol binding protein (CRBP), metabolized into retinaldehyde (RAL), which is irreversibly converted into RA. Intracellular RA is transported by cellular retinoic acid binding proteins (CRABP) or fatty acid binding protein 5 (FABP5) and it can be degraded by CYP26 or translocated to the nucleus, where it binds and activates nuclear receptors. If transported by CRABP it binds nuclear retinoid acid receptors (RARs), whilst in association to FABP5 it binds peroxisome proliferation-activated receptor β/δ (PPARβ/δ), activating the transcription of specific target genes; (**B**) RA extranuclear effects. In response to RA, a subpopulation of RARα and RARγ present in membrane lipid rafts activates kinase cascades. In neuronal cells, extracellular signal-regulated kinase (ERK) phosphorylation is mediated through RARγ in association with sarcome (Src) kinase, whereas in other cellular subtypes RARα is the effector of the transduction cascade through p38 mitogen-activated protein kinase (p38MAPK) or ERK signalling. Activated p38MAPK and Erks translocate to the nucleus where they phosphorylate several targets, being mitogen- and stress-activated protein kinase (MSK1) a good candidate. Plasma ROH, bound to RBP4, binds to the cell receptor STRA6 which phosphorylates and activates Janus kinases2/signal transducers and activators of transcription 5 (JAK2/STAT5) signalling pathway. This phosphorylated STAT5 translocates into the nucleus where it regulates gene expression of target genes.

Retinal, the oxidized form of retinol, plays a key role in vision being the precursor of the visual chromophore 11-*cis*-retinal. In vertebrates, phototransduction is initiated by a photochemical reaction where opsin-bound 11-*cis*-retinal is isomerized to all-*trans*-retinal. The photosensitive receptor is restored via the retinoid visual cycle [47,59]. On the other hand, most of the physiologic actions of retinoids are accounted by the ability of RA stereoisomers to bind nuclear retinoid receptors, which function as transcription factors modulating the expression of several hundred genes [12]. From many studies, it can be concluded that RA is involved in the regulation of more than 500 genes. In some cases, the control of gene expression is exerted by RARs directly, mainly by direct binding of RAR/RXR heterodimers to RAREs on the promoter of responsive genes. However, in some cases, gene regulation is achieved through an indirect action of RARs onto responsive genes (Figure 1). At the moment, two families of nuclear receptors, RA receptors (RAR isotypes α, β and γ, also referred to as NR1B1, NR1B2 and NR1B3 and their isoforms) and retinoid X receptors (RXR isotypes α, β and γ, also referred to as NR2B1, NR2B2 and NR2B3 and their isoforms) are described. RARs act by forming heterodimers with RXRs, whereas RXRs can form either homodimers or heterodimers with several partners including RARs, vitamin D receptor, PPAR, thyroid hormone receptor and orphan nuclear receptor. All-*trans* RA, the predominant isomer in vivo and 9-*cis* RA activate RARs, whereas RXRs are activated by 9-*cis* RA. However, the endogenous presence of 9-*cis* RA has never been rigorously confirmed and the consideration of this compound as an endogenous RXR ligand (rexinoid) is a controversial matter. Recently, an alternative ligand (9-*cis*-13,14-dihydroretinoic acid) has been proposed as a physiologically relevant rexinoid in mammals, although additional work is required to determine its expected role in mammalian signalling [60]. The RAR/RXR heterodimer binds weakly to specific DNA sequences, called RA response elements, RARE and located within the regulatory region of target genes. RARE are characterized by two direct repeats of the core hexameric motif 5′-(A/G)G(G/T)TCA-3′ separated by differently sized nucleotide motifs. When activated by a retinoid, the heterodimer binds tighter to RARE, releases corepressors, recruits coactivators and the transcription machinery, and initiates gene transcription. In the absence of a ligand, DNA-bound RARs associates with corepressors, which recruit protein complexes with histone deacetylase activity. By removing acetyl residues of histones, these complexes maintain chromatin in a condensed, repressed state. RARs and RXRs are actively regulated by post-translational modifications, which mainly include phosphorylation processes of specific and conserved serine residues induced by retinoid activated kinases. Phosphorylation significantly affects the binding of RAR to DNA, co-regulators recruitment and degradation. Nevertheless, the mechanism and the biological role of these events in the pleiotropic action of retinoids are currently unstated [10,47,61,62,63].

Several studies over the last decade have suggested that RA displays biological activities that are independent of its ability to activate RAR. RA can also function as an agonist for a different nuclear receptor, namely PPARβ/δ (also referred to as NR1C2). PPARs, like RARs, interact with RXR to form heterodimers which when are activated by its ligand bind to PPAR response elements, PPRE, in regulatory regions of specific genes to induce target gene transcription. PPARβ/δ is involved in keratinocyte differentiation, neuronal development and inflammation and, like other PPARs, is also involved in lipid metabolism and insulin resistance. RA signalling through RXR: PPARβ/δ has acquired a great interest for energy homeostasis and insulin response. Partitioning RA between RARs and PPARβ/δ is governed by different intracellular lipid-binding proteins: CRABP2 selectively delivers RA to nuclear RARs and a fatty acid binding protein, FABP5, delivers RA from the cytosol to nuclear PPARβ/δ. Consequently, since RARs and PPARβ/δ regulate the expression of distinct sets of genes, RA stimulates different cellular responses depending on whether RARs or PPARβ/δ are activated [64,65].

Furthermore, besides classical nuclear receptor signalling, RA stimulates extra-nuclear non-transcriptional effects through the rapidly and transiently stimulation of kinase signalling pathways by extra-nuclear RARs which also can affect gene transcription. Depending on the cell type, the extra nuclear effects of atRA appear to involve different mechanisms and kinase cascades. In line with this concept, though classically known to reside in the nucleus, RARs have been reported to be present in membranes. Indeed, it has been shown that a subpopulation of RARs (RARα or RARγ) is present in membrane lipid rafts and activates kinase cascades in response to atRA. In various epithelial and fibroblast cells, atRA activates p38 mitogen-activated protein kinase, p38MAPK, through the interaction of RARα with Gαq. In neuronal, sertoli and embryonic stem cells atRA activates p42/44MAPK (also called extracellular signal-regulated kinase, Erks) via RARα and phosphoinositide 3-kinase, PI3K, or via RARγ and the sarcome, Src, kinase. The activated p38 and p42/44MAPKs translocate to the nucleus where they phosphorylate several targets. One of these targets is mitogen- and stress-activated protein kinase, MSK1. Both MAPKs and MSK1 also phosphorylate histones and several nuclear proteins involved in the transcription of the RA-target genes, including RARs themselves and their co-regulators [10,62,63]. Recently, it has been shown that RA modulates glucocorticoid receptor signalling in the nucleus of a hippocampal HT22 cell line by increasing its phosphorylation in Ser220. This effect is mediated at least in part via the cyclin dependent kinase 5, CDK5 and its cofactor p35 which may participate to the beneficial effect of RA on neuronal cells in addition to other mechanisms [66].

Moreover, it has become increasingly evident that, in addition to RA, others vitamin A derivatives, among them oxoretinoids, are also biological active vitamin A compounds. Interestingly, for example, all-*trans*-4-oxoretinol, first considered to be an inactive metabolite of RA, has been identified as a physiologically retinoid signalling molecule that exerts an important biological activity and regulates some of the same genes as RA, independently of its intracellular transformation into RA [67,68]. Furthermore, nuclear RARs are targets for *S*-4-oxo-9-*cis*-13,14-dihydro-RA that activates the transcription of RARE-containing genes in several cell types both in vitro and in vivo. This oxoretinoid was reported to be present in liver and other tissues of experimental animals, as well as in humans. These compounds and their synthetic analogues are emerging as promising candidates with a significant therapeutic potential [60,69,70].

Additionally, new concepts are now arising and retinol have proved to be active and also to stimulate kinase pathways, resulting in the activation of other subsets of genes involved in lipid homeostasis and insulin responses, increasing again the spectrum of action of retinoids. As mentioned before, retinol bound to RBP4 (holo-RBP) activates intracellular signalling pathways. The binding of the retinol-RBP complex to STRA6 not only induces retinol transport into cells but also triggers the phosphorylation of its cytosolic domain which activates the JAK/STAT signalling cascade. As a result, STAT target genes, such as SOCS3 (suppressor of cytokine signalling 3) which inhibits insulin signalling and PPARγ, which enhances lipid accumulation, are up-regulated. Hence, holo-RBP functions like a classical cytokine to activate a STRA6/JAK2/STAT3/5 pathway [55,71]. Furthermore, the work by Ziouzenkova et al. [52] shows that retinaldehyde, the natural metabolite of retinol oxidation, is a signalling molecule in fat tissue, with its own distinct effects independent of RA formation via aldehyde dehydrogenase-1 family of enzymes and the key role in the retina for night vision. At the molecular level, retinaldehyde modulates adipogenesis by inhibiting the activation of RXRs and PPARγ. The intracellular carrier proteins—CRBP and CRABP—for retinol, retinaldehyde and RA are not just passive carriers but instead contribute to the overall regulatory network [13,52,72].

Consequently, the pleiotropic effects of vitamin A might be due to the diversity of the several natural retinoids with different biological activity, their diverse action mechanisms and the variety of the participants implicated.

## 3. Vitamin A Deficiency

### 3.1. Epidemiology and Incidence

Plasma retinol levels are typically measured to assess vitamin A status; however, their value is limited because plasma retinol levels are under tight hepatic homeostatic control and do not decline until vitamin A concentration in the liver is almost depleted (critical liver concentration ≤ 20µg g^−1^ of liver [40]. Liver vitamin A reserves can be measured indirectly through the relative dose-response test [41], which is considered the “gold standard” indicator of whole-body vitamin A status; however, for clinical purposes, plasma retinol levels alone are sufficient and commonly used for documenting significant deficiency of vitamin A [73,74]. The physiological plasma concentration of vitamin A is 1–2 µmol/L and according to the World Health Organization, values of serum retinol concentrations below a cut-off of 0.70 µmol/L (or 20 µg/dL) represent biochemical VAD and values lower than 0.35 µmol/L are indicative of severe deficiency and associate with numerous clinical manifestations [15,24].

VAD is a major public health problem in more than half of all countries. This deficiency constitutes, together with protein malnutrition, the most common nutritional disorder in the world; in fact, it is estimated that 250 million preschool-aged children in developing countries have biochemical VAD and 5 million are clinically affected by this deficiency. The latest WHO global estimations of VAD (1995–2005) reveal that, based on the prevalence of plasma retinol concentrations below 0.70 µmol/L, 122 countries have VAD of public health significance. In these countries, along with preschool children, other groups of people at high risk for VAD are pregnant and lactating women [15,24]. The deficiency is endemic in developing countries, particularly in Africa and South-East Asia, mostly because residents have limited access to foods that contain preformed vitamin A from animal-based food sources and do not commonly consume available foods containing β-carotene, which are abundant in relatively expensive vegetables and fruits.

VAD is rare in developed countries where vitamin A intake is higher in comparative terms; however, it is important to note that over 20% of the population in the developed world does not reach two-thirds of the recommended intake and has plasma and liver concentrations of vitamin A lower than those accepted as normal. In this context, higher prevalence is found in the poverty areas of the developed communities and may result important among pregnant women of lower social status and in adults or children affected by human immunodeficiency virus. This situation can be aggravated by the increasingly common tendency to reduce fat intake and to engage in uncontrolled weight loss diets (exacerbated in psychiatric eating disorders e.g., anorexia and bulimia) and vegetarian culture. Other risk factors for VAD include stress, diseases which affect the intestine’s ability to absorb fat, obesity and also bariatric techniques for its treatment, infections, infestations, alcohol abuse and interactions with other xenobiotics which could disrupt normal retinoid homeostasis. Excessive alcohol consumption results in a reduction of liver vitamin A reserves due to lower consumption of foods, competitive inhibition of retinol oxidation, mobilization of the vitamin A from liver and the induction of enzymes which degrade retinol and RA. The hepatic depletion was strikingly exacerbated when ethanol and drugs (that induce cytochromes P450 in liver microsomes) were combined, which mimics a common clinical occurrence [19,20,21,24,39,43,75,76]. Intestinal infestations and severe infections in adult patients (i.e., sepsis and pneumonia) result in excretion of large quantities of retinol in the urine and depletion of vitamin A stores [77,78]. Retinol levels decline rapidly as part of the acute phase response [79] and this translates into increased susceptibility to infection, creating a “vicious circle” difficult to break [80]. Therefore, in all of these situations it is important to consider the subclinical deficiency which has increased dramatically worldwide in the last decades [17,18,24].

The WHO’s goal is the worldwide eradication of VAD and its tragic consequences, including blindness, disease and premature death. Over the last decade global improvement in VAD control has been achieved, by promoting breastfeeding, intensifying the distribution of supplements, fortification of foods and through horticulture and education programs [15].

### 3.2. Clinical Manifestations and Tissue Damage

Accordingly, with the multiple functions of vitamin A and its biologically active derivatives, VAD has a remarkable number of clinical manifestations, ranging from xerophthalmia (dryness, thickening and loss of transparency of the eye conjunctiva), practically pathognomonic, and nyctalopia (night blindness) to dry skin, metaplasia and keratinization of mucosal epithelial surfaces. This leads to clinical abnormalities of conjunctival and corneal xerosis, as well as epidermoid metaplasia and other epithelial defects throughout the respiratory, genitourinary and gastrointestinal tracts and glandular ducts, disturbances in cell differentiation, organ development and growth and increased susceptibility to severe infection. VAD is the leading cause of preventable blindness in children and increases the risk of disease and death from severe infections.

The term “vitamin A deficiency disorders” (VADD) has been coined to cover the whole clinical spectrum of disease [15,17,18,41]. Night blindness is the first symptom of the deficiency and is accompanied by degenerative changes of the retina and dryness of the conjunctiva (xerosis), which produces a greyish pigmentation called Bitot spots. It is estimated that 250,000 to 500,000 vitamin A-deficient children become blind every year, half of them dying within 12 months of losing their sight [15]. The control of blindness in children is closely linked to child survival and reducing its prevalence is one of the goals of WHO by the year 2020 (World Health Organization’s VISION 2020: The Right to Sight Programme (Gilbert and Foster 2001; https://www.iapb.org/vision-2020/).

Classic studies in multiple species, including hamsters, rats and mice, have shown major pleiotropic effects of maternal dietary vitamin A deprivation in embryonic development. Many organs and systems are affected by VAD during the stage of the prenatal development or in the postnatal life. Foetal resorption is common in severe VAD, growth fails, and vascularization stops, while foetuses that survive have characteristic malformations of the eye, lungs, urogenital tract and cardiovascular system. This situation could be reverted by re-feeding vitamin A-deficient animals with retinol, thus confirming the involvement of vitamin A in the maintenance of organ morphology and cell differentiation [16]. Similar abnormalities leading to foetal death or to morphological malformations are also observed in rat embryos lacking nuclear retinoid receptors and/or altered retinoid signalling [81,82,83]. Therefore, it is clear from both nutritional and genetic studies that retinoids, through RA signalling, play a critical role in many stages of embryogenesis and this is accomplished through the precise regulation of RA synthesis and catabolism via the RALDH and CYP26 enzymes, although it seems insufficient to support gestation of the embryo. For a more detailed discussion of the different roles of vitamin A during embryogenesis, see the following reviews [84,85,86,87,88,89,90].

Available evidence showing that nutritional VAD induces negative histologic changes in different tissues indicates that retinoids continue to play an important role in the postnatal life. VAD during the growing postnatal period in rats induced alterations in the mucous epithelium leading to cornea ulcerations, squamous metaplasia, necrotizing tracheobronchiolitis, altered immune function, increased risk for cancer and sterility. Chronic VAD was also associated to changes in the morphology and the ultrastructure of ECM in kidney, lung and liver, which is related to fibrogenic activation and deterioration of tissue parenchyma. Other adverse effects such as hepatic steatosis, decreased protein metabolism, pulmonary stress and oxidative damage to liver mitochondria, decreased respiratory complex in heart and altered expression pattern of p53 and proliferative control genes in liver and lung have also been described. Once vitamin A levels and retinoid signalling were restored to normality, these changes were almost reversed, thus confirming the involvement of vitamin A in the preservation of organ architecture and cell differentiation in the growing period and providing a justification for the therapeutic use of retinoids and vitamin A supplementation programs [32,34,91,92,93,94,95,96,97,98,99,100,101]. In fact, RA (isotretinoin) and vitamin A are currently valued in more than 400 clinical trials in the treatment of different diseases, including cancer, kidney pathologies, emphysema and other lung diseases, and, among others, in improving the lipid profile (https://clinicaltrials.gov/ct2/results?term).

Vitamin A also interplays with endocrine tissues and hormonal systems, that is, VAD provokes thyroid dysfunction. Recently, it has been described that maternal VAD affects foetal pancreatic islet vascularization and development and that retinoids are needed also by the adult to assure normal pancreatic endocrine functions, especially those of the α- and β-cells. VAD affects pancreatic progress and function causing apoptosis of pancreatic beta-cell masses which can be reverted by RA administration [102,103,104]. Also, altered retinoid signalling has profound effects on several physiological and pathological processes in the brain. RA signalling is widely identified in the adult central nervous system, including amygdala, cortex, hypothalamus, hippocampus and other brain areas. VAD and knockout mutations of RAR have been shown to impair spatial and working memory of rodents, thus providing evidence that vitamin A status affects cognitive ability. In this sense, VAD was associated with apoptosis in the hippocampus and decreased neurogenesis. Moreover, adult humans with VAD or deficient RA signalling show defective performances in spatial learning and memory tasks and, in parallel, this deficiency could both induce amyloid β protein (Aβ) overproduction and inhibit Aβ protein clearance by glial cells, leading to Aβ accumulation and increased susceptibility to Alzheimer’s disease [105,106,107,108,109,110,111].

Several organs and systems are affected by VAD but the developing lung is especially sensitive to changes in the levels of vitamin A. Retinoids play a key regulatory role in lung from embryogenesis to adulthood. Keratinization of the tracheae and bronchi appears to occur very soon in deficiency, preceding alterations in the eyes and predispose to the development of infection [9]. VAD during pregnancy or RARα and β double null mice is known to result in lung hypoplasia and lung agenesis of the embryos [26,29,90,112,113]. Several evidences also support that retinoids play an essential role in adult life. Vitamin A is essential in the formation of lung alveoli, which constitute the gas exchange region of the lung, which takes place during pregnancy and continues for several years after birth. Chronic nutritional VAD results in decreased alveolar septation and in marked changes in the respiratory epithelium. Moreover, chronic VAD has been linked to lung functional defects and disease states both in human and in animal models [23,28,29,58,114,115,116].

In this review, we will summarize our current knowledge regarding the link between VAD and several lung pathologies, through foetal lung differentiation and maturation to postnatal state and disease progression. Many respiratory diseases have been associated with vitamin A status. One major focus has been the link between VAD and childhood asthma. VAD has been associated with an increased risk of respiratory infections. Other chronic respiratory diseases associated with VAD include emphysema, chronic obstructive pulmonary disease (COPD), pulmonary fibrosis and lung cancer. We will also show our recent findings on morphologic alterations in the BM proteins and in the modification of cell-junction proteins in VAD lungs and briefly provide an overview of the potential pathways involved in the pathogenesis of these diseases, which collectively represent a major global health problem constituting one of the leading current causes of death worldwide [35].

## 4. Vitamin A Deficiency and the Lung

### 4.1. Overview

Human lung development begins in the fifth foetal week and continues throughout the first few years of life. It includes several phases: embryonic, pseudoglandular, canalicular, saccular and alveolar phase. The foetal lung develops postnatally to become one of the most complex organs, characterized by approximately 40 different cell types. The primary function of the lung is to meet the organism’s need for oxygen and CO_2_ removal. This takes place in lung alveoli which are formed in part by subdivision (septation) of the gas-exchange saccules of the immature lung. The development stage during which septation occurs varies considerably among species. In humans, the timely process of lung differentiation to form alveolar structures occurs mainly during the third trimester of pregnancy and continues in postpartum, over the first years of life. During perinatal period, lung maturation proceeds in a highly controlled process, where alveolar epithelial/mesenchymal interactions play a key role. However, in rats, the alveolar phase does not begin until postpartum and slowly develops throughout life. Because of this delay in lung maturation, rats have been used as an animal model to study human lung diseases of the preterm neonate [112,117]. Pulmonary alveoli are the functional units of the lungs in the process of breathing. Results found in RAR null mice indicate that the different RARs play distinct roles during alveoli formation. RARγ could be a positive regulator and RARβ could be a negative-regulator of alveolar septation. RARα seems to be required for post-natal alveolar regeneration [63].

Alveolar type II pneumocytes are in direct contact with the alveolar type I pneumocytes above the BM and fibroblasts and other interstitial cells below the membrane. Before parturition, fibroblasts secrete a polypeptide that stimulates the rate-limiting enzyme for surfactant synthesis by the alveolar type II pneumocytes. In addition, the type II cell serves as a progenitor for the type I pneumocyte, which is the major resident of the alveolar wall and is therefore important for normal lung maintenance. Surfactant, a lipoprotein that reduces surface tension preventing the pulmonary collapse during the respiration, also participates in host defence and inflammation response in the lung. RA is able to control the expression of surfactant protein in human foetal lung explants. Insulin, transforming growth factor β (TGF-β) and glucocorticoids can also modulate the expression of surfactant protein [28,117,118].

As described above, vitamin A is stored in hepatic stellate cells in liver but also in other organs. These cells, which have been also detected in the lungs including foetal lung, can take up retinol from chylomicron, suggesting that this organ acquires it by a similar mechanism as liver, although its concentration is considerably lower than in the liver. Total vitamin A (free plus esterified) concentration in human lung of subjects ranging in age from 4 months to 86 years varies between 8.7–1102.2 nmol/g tissue in liver and 0.7–404.6 nmol/g tissue in lung [119]. The main role for RE stores in the lung is to ensure direct retinol delivery when there is an increased retinol demand by this tissue, especially in the developing lung, when the morphology of the lungs is still immature. Thereby, maternal vitamin A supply is of essential importance for adequate foetal supply, growth and development. These stores are the basis for RA synthesis during lung maturation and post-natal function. Moreover, the foetal/neonatal synthesis of RBP is not sufficient to ensure continuous supply from liver stores. Inadequacies in the nutritional requirements of the developing lung in utero compromise the respiratory system integrity. Vitamin A, through the related formation of RA, is important in regulating early lung development and alveolar formation. Therefore, sufficient intake should be ensured during the last month of pregnancy, assuring retinyl stores in the developing lung, essential for RA synthesis during lung maturation and postnatal life. During embryonic development, RA regulates cell proliferation and differentiation and regular organogenesis. VAD during pregnancy or RARα and β double null mice is known to result in altered lung morphogenesis of the embryos and results from the different RAR double mutants implicate both RARα and RARβ as critical receptors for the developing lung. Moreover, a balanced activation of both receptors is critical for appropriate lung bud initiation and endodermal differentiation. It has been concluded that RA co-ordinately regulates several endogen pathways including wingless-Int (Wnt), bone morphogenetic protein (BMP) and TGF-β signalling to modulate fibroblast growth factor 10 (Fgf10) expression which is a crucial factor for the induction of lung buds [26,29,90,112,113,118,120]. Low vitamin A status of the new-born appears to contribute to the risk of bronchopulmonary dysplasia (BPD), a chronic lung disease with focal loss of ciliated cells with keratinizing metaplasia and necrosis of the bronchial mucosa as well as increased mucous-secreting cells, and this acquires more importance in premature infants where serum retinol and RBP levels are significantly lower than in full-term neonates. Moreover, mature lung function might be a consequence of adequate alveolar formation during foetal lung development and during early childhood. In fact, low neonatal liver stores and a low supply during lactation have been linked with abnormal lung function, decreased alveolar number, reduced protection against infections and higher probability of development of acute illnesses in childhood and chronic illnesses in adulthood, including the risk of lung cancer [23,118,121].

Additionally, in the postnatal period, RA is essential for lung growth, alveolarization and expression of the major components of the ECM which plays a main role in resistance and elasticity, repair and remodelling of lung. Consequently, VAD will induce profound changes in lung architecture and function. In humans, endemic VAD is associated with a low forced vital capacity (FVC), an indicator of airway obstruction and a strong predictor of mortality in asymptomatic adults without chronic respiratory conditions. Interestingly, in a recent work, a new gene associated with FVC, NCOR2 (nuclear receptor corepressor 2), also known as SMRT (silencing mediator of retinoid and thyroid hormone), which is implicated in the RA signalling pathway has been identified and this emphasizes again the importance of vitamin A metabolism in the regulation of lung growth and maintenance [122].

It was as early as in 1913, when McCollum, who described “fat-soluble A” which was later identified as retinol, observed that the animals made deficient of this factor “have frequently suffered from prevalent bronchitis”. Moreover, as has been mentioned above, keratinization of the tracheae and bronchi appears to occur very soon in VAD, indeed preceding alterations in the eye [8,118,120,121,123]. Since then, numerous reports have shown that VAD is associated with several lung diseases. However, in this review we will consider those that have the greatest impact and where the relationship has been better established.

### 4.2. Respiratory Infections

Respiratory infections are a major cause of morbidity and mortality worldwide [35]. Acute respiratory infections—mainly pneumonia and influenza—result in over 4 million deaths worldwide each year. They are the leading causes of illness and death among children under 5 years of age. About 1.3 million children die from acute respiratory infections worldwide, constituting one third of the deaths in under five in low income countries and remaining the leading causes of paediatric death worldwide. Moreover, in a global context, the death rate from these infections alone is 10 times higher than the global median death rate from all causes [124,125,126].

Hopkins, McCollum and Osborne and Mendel [8,127,128] found that animals fed only fats, protein, starch and inorganic salts failed to grow normally, showed increased susceptibility to infection and often died of overwhelming sepsis. In 1928, Green and Mellanby had confirmed vitamin A as an anti-infective factor [129]. Several studies, confirm that vitamin A is an immune-modulating agent and plays an important role in the immunological response to infections [99,130,131]. In accordance with these results, VAD markedly contributes to childhood morbidity and mortality being the epithelia of the trachea and respiratory tree among the first tissues to show histological changes. Earlier works of Sommer and colleagues showed that ‘‘the risk of respiratory disease and diarrhoea were more closely associated with vitamin A status than with general nutritional status’’ [22]. The epithelial tissues are the first barrier of defence from pathogens in animals. Many of the effects of undernutrition are mediated through the immune system, including changes in host defences that affect resistance to or recovery from infections. VAD causes squamous metaplasia of the respiratory epithelium, where the ciliated epithelial cells are replaced by squamous epithelium and also provokes a decrease in mucus production. These are factors that can increase the risk of invasive pathogens. The function of residents macrophages, neutrophils and natural killer cells and the development of T-cells mediated antibody responses are also impaired in VAD, leading to a decreased protective mechanism at mucosal surfaces [91,98]. During embryogenesis, maternal RA is essential for the development of secondary lymphoid organs [132], whereas throughout the adult life RA controls the differentiation of immune cells necessary for immune tolerance via Treg induction in in vitro and in vivo animal models. In addition, evidence for effects of vitamin A on epigenetic regulation of immune function is emerging [130].

In individuals with reduced plasma vitamin A levels, repeated respiratory infections are more frequent and this constitutes one of the main health problems in developing countries. In addition, during infectious diseases and particularly of the respiratory tract, plasma retinol levels decline and this induces an increased susceptibility to infection creating a “vicious circle”. This can be explained with an increased metabolic demand and/or an increased renal elimination of retinol and of RBP during acute infections [23,80]. Vitamin A supplementation is administered to infants in developing countries and is generally accepted that supplementations are beneficial and reduces respiratory infections but this is not always the case and supplementation is still controversial [18,25,99,133,134]. On the one hand, vitamin A supplementation among individuals at high risk of tuberculosis also seems to be effective in preventing this disease which remains actually the tenth leading cause of death in the world and one of the top causes of death from infectious disease, the vast majority occurring in low- and middle-income countries [35,135,136]. However, others supplementation trials found no evidence for a beneficial effect in areas with a high prevalence of VAD. Perhaps conflicting clinical results can be explained, at least in part, by the distinct response of supplementation against some pathogens (i.e., it could be favourable to diminish diarrheal disease but not respiratory infections), or its differential effects depending on the nutritional status (its administration is protective against infection in malnourished children but could be detrimental for well-fed children), or the most recent vaccines given, the sex and age of the child, the season or the differential effects of RA on target cells [25,130,134,137,138,139,140].

### 4.3. Asthma

Asthma is a chronic inflammatory disease characterized by a nonspecific hyperirritability of the tracheobronchial tree with variable airway inflammation, reversible airflow obstruction, bronchial hyper responsiveness (increased propensity in airway constriction in response to bronchoconstricting stimuli) and recurrent episodes of wheezing and coughing. The aetiology of airway hyper responsiveness in asthma is unknown, may be increased by a number of factors (i.e., allergenic, infectious, exercise-related) and airway inflammation plays a fundamental role. Asthma often presents with clinical features such as airway infiltration of mast cells, eosinophils and activated T helper lymphocytes. It currently affects approximately 235 to 300 million people in the world and is expected to affect another 100 million people by 2025; in fact, its prevalence is increasing worldwide as communities adopt modern lifestyles and become urbanized, maybe through increasing obesity, interactions with common environmental microorganisms and exposure to open-air pollution and allergens. Asthma results in approximately 200,000 deaths worldwide every year, mostly in low- and middle-income countries and is found frequently in the paediatric population. Several randomized and controlled trials provide strong evidence to support the treatment guidelines of both the Global Initiative for Asthma and the Expert Panel Report 3 of the National Asthma Education and Prevention Program, which recommend the use of corticosteroids and long-acting β2-agonists (LABA) for asthma treatment, as well as the recent FDA decision to remove the safety warning of this combination. A lower consumption of antioxidant vitamins can promote higher vulnerability to oxidative stress and increased susceptibility to suffer from asthma; systematic reviews show evidence of a beneficial effect of fresh fruits and antioxidant vitamins on prevention and treatment of asthma [125,141,142,143,144,145].

An inverse relationship between vitamin A status and the degree of airway obstruction, assessed by forced expiratory volume in one second (FEV1), has been established in humans [116]. Several observational studies reported that VAD is associated with a higher risk of asthma and severe wheezing. In addition, an inverse correlation between VAD and bronchial hyper reactivity in experimental animals has been proposed. Different mechanisms can be postulated to explain the effects seen in vitamin deficiency. VAD provokes altered ciliated columnar epithelial cells, squamous metaplasia and decreased defensive ability of glandular cells in the respiratory tract which could lead to an increased risk of suffering from asthma. Oxidative stress associated with increased reactive oxygen species (ROS) and/or reactive nitrogen species may act as mediators of the molecular and cellular events implicated in the pathogenesis of asthma by increasing the release of pro-inflammatory cytokines; in this sense, it is known that VAD is a condition which leads to an imbalance between ROS production and antioxidant defences in lung [32,146,147,148,149,150]. Moreover, the airway hyper reactivity in VAD rats has been associated to a diminished ability of muscarinic M-2 receptor mediated suppression of bronchoconstriction, because there is a reduction in the expression of muscarinic M-2 receptor in their bronchial tissue compared with control rats. This effect can be reversed with RA. Furthermore, a similar reduction in muscarinic M-2 receptor function has been observed in asthma. Other studies suggested that, at least in part, the airway hyper responsiveness can result from essential changes in the bronchial smooth muscle phenotype and provide evidence that endogenous RA plays a key role in controlling the airway bronchial smooth muscle differentiation program during the airway development. Moreover, alterations in lung ECM which are present in asthma, as bronchial BM thickening, are similar to those observed by our group in an experimental model of chronic VAD. These changes, which are probably mediated by TGF-β1, were almost totally reversed by RA [33,113,114,130,151,152,153,154,155].

Increased serum vitamin A induced a good pulmonary function and a good quality of life in children with stable asthma; also, RA reverses airway hyper responsiveness associated with VAD in rats and might protect from asthma by downregulation of oxidative stress or direct effects on the immune system. However, other studies concluded that vitamin A supplementation early in life was not associated with a decreased risk of asthma in an area with chronic VAD. The multifactorial origin of conditions that lead to hyper responsiveness and asthma indicate that additional factors, such as pulmonary infections or airway inflammation, should not be ignored in the pathogenesis of these diseases; moreover, an inverse association between asthma risk and vitamin A status could be explained by a reduction in serum retinol plasma levels due to inflammation. Thus, currently, there is not sufficient evidence for the strategic use of retinoids in treating these diseases [113,130,143,145,148,152,156,157].

### 4.4. Emphysema and Chronic Obstructive Pulmonary Disease

Pulmonary emphysema is a common disease in which destruction of the alveolar inner walls leads to larger but fewer alveoli, decreased surface area for gas-exchange air and inadequate oxygenation. It commonly associates with chronic bronchitis, a condition with chronic cough and phlegm. Emphysema and chronic bronchitis comprise the disease known as chronic obstructive pulmonary disease (COPD), which is characterized by airflow limitation, gradual loss of lung recoil and long-term breathing problems. Consequently, maximal expiratory flow is reduced. COPD is a progressive disorder even when contributing factors are removed and therapy is established; progression is inevitable, since loss of elastic tissue is a normal part of the aging process. Thus, treatment may slow the progression of COPD but it cannot reverse the damage. COPD prevalence and mortality is of increasing public health importance. An estimated 210 million people suffer COPD worldwide. This disease claimed 3.0 million lives in 2016 being currently one of the top three causes of mortality [35]. It has been established that cigarette smoking is the leading cause of COPD and smoking cessation reverts the rate of decline FEV1, although lost lung function is not regained [25,58,158,159].

The mechanisms through which emphysema develops are not satisfactorily known and several possibilities may be considered. First, an increase in elastase activity could be involved leading to a decrease in matrix protein elastin which seems to be related to the development of emphysema. Second, the imbalance of oxidant/antioxidant supply could induce oxidative injure to the tissues leading to emphysema. Both mechanisms are suggested to be present during exposure to cigarette smoking, the most linked cause of emphysema [58].

Vitamin A and its active metabolite, RA, influence alveolar development and tissue repair [26] and it has been demonstrated that VAD induces emphysema [58]. The biochemical mechanism underlying VAD and emphysema has intended to be explored in basis of the two mechanisms proposed for the development of this respiratory disease. In weanling rats fed a vitamin A-deficient diet alveolar septation is significantly reduced and the lungs show areas with emphysematous features such as increased size of air spaces distal to the terminal bronchiole with thinning and partial or total destruction of the septal wall. These alterations in lung function and architecture are associated with modifications in ECM/BM. During alveologenesis, it is known that ECM interacts with fibroblastic, epithelial and microvascular cells. Destruction of lung matrix, especially elastin, results in emphysema. In murine lung, VAD during foetal development leads to quantitative changes in elastin and collagens and these are involved in the defects of alveolarization and lung function deficiency induced by vitamin deficiency [28,30,58,160,161]. In a model of chronic VAD rats we have shown that vitamin deficiency during the growing period also results in emphysemic lungs, which associates with alterations in ECM/BM and an increase in TGF-β levels in pulmonary tissue. The BM doubled its thickness and its component macromolecules, such as collagen IV and laminin, are also modified, not only quantitatively but also qualitatively in VAD rat lungs [32]. The relationship between retinoids and the TGF-β system appears quite complex and is not clearly stablished. It is known that VAD is a condition which leads to an imbalance between ROS production and antioxidant defences in lung [32,91]. ROS, acting as secondary intracellular messengers, have been shown to activate transcription factors, such as activated protein-1 (AP-1) and to induce the synthesis of the fibrogenic cytokine TGF-β1 and of various ECM proteins. TGF-β1 in paracrine action also induces the expression of type I and type IV collagens in fibroblasts and epithelial–endothelial cells [162,163,164]. Although many cell types are able to synthesize TGF-β1, data from our previous studies in VAD animals lead us to proposing the inflammatory cells as its main source in lung tissue [32]. Accordingly, experimental data suggest that oxidative stress plays a key role in the pathogenesis of many inflammatory lung disorders such as asthma, COPD, idiopathic pulmonary fibrosis (IPF), cystic fibrosis and adult respiratory distress syndrome. In experimental models of emphysema, RA prevented the decreased number of alveoli and stimulated alveolar wall formation when administered postnatally. Also in our studies, RA partially reverses the alterations found in the BM thickness and ultrastructure in VAD rat lungs. All these studies suggest that vitamin A/RA could be beneficious for the prevention or treatment of emphysema. Despite promising results in experimental animals, randomized controlled with placebo studies in patients yielded disappointing results, perhaps due to the remarkable differences in the pathophysiology of human emphysema [32,33,58,63,159,165,166,167]. Recently, a new mechanism has been suggested whereby RA signalling can regulate alveolar maintenance and repair in adult human lungs through microvascular angiogenesis, which improves alveolarization. On the contrary, degradation of endogenous RA by increased CYP26A1, which occurs in emphysema, impairs endothelial cell repair and may contribute to chronic lung disease. This supports the clinical importance of maintaining an adequate vitamin A status for the conservation of lung function in humans [168].

As previously mentioned, the most linked cause with emphysema is cigarette smoking. Exposure to tobacco smoke leads to oxidative stress, increased mucosal inflammation and increased expression of inflammatory cytokines and tumour necrosis factor (TNF)-α and these factors play a key role in an impaired lung function and are involved in lung diseases. Moreover, the emphysema caused by smoking cigarettes also may be provoked by a deficiency of local vitamin A of the lungs. Rats exposed to cigarette smoke showed a decrease in vitamin A levels in serum, lung and liver, which were associated with areas of emphysema and inflammation in lung similar to that seen in VAD lungs. Furthermore, adult ferrets exposed to cigarette smoke revealed an increased catabolism of RA and presented lower levels of RA in their lungs. The relationship between cigarette smoking and vitamin A levels in humans is less clear than in experimental animals; however, there are several studies which indicate an inverse relationship between cigarette smokers and vitamin A status in adult population. In this context, it is important to point out that alcohol abuse which is known to induce vitamin A depletion and tobacco smoking are conditions commonly associated in adult population [36,58,169]. Accordingly, a higher vitamin A and β-carotene intake is proposed in patients suffering from lung diseases such as COPD, however with caution, since the elevated intake of β-carotene and retinol could increase the incidence of lung cancer and cardiovascular disease mortality (see Section 4.5) [58,150,169,170,171,172].

### 4.5. Lung Cancer

Vitamin A plays a main role in regulating antioxidant defences, cell growth and differentiation. Consequently, numerous studies have focused on the association between vitamin A and various types of cancer, although the relationship between serum vitamin A status and cancer risk is still unclear [160,172]. Lung cancer is a leading cause of cancer mortality worldwide. It has been suggested that inflammation plays a key role in the pathogenesis of lung cancer and that pulmonary disorders such as COPD and emphysema, constitute comorbid conditions and are independent risk factors for lung cancer [173]. It has been shown that among all the modifiable factors, smoking interruption plays an important role in the prevention of decreased lung function and lung cancer incidence. With regard to vitamin A, a recent meta-analysis indicated that the highest category of dietary vitamin A intake could reduce the lung cancer risk compared with lowest vitamin A category [174]. It has been shown that the incidence of several epithelial tumours increases in humans with low levels of plasma retinol as well as with a low vitamin A dietary intake. In agreement with those results, VAD in rats augmented the epithelial thickness increase and mucosal cell hyperplasia caused by cigarette smoke [175]. In human lung cancer cell lines, RAR-β transfection decreases the rate of cellular proliferation and also the tumorigenicity after inoculation into mice [176]. Moreover, the risk of death from lung cancer was reduced by 40%–50% in participants receiving β-carotene, α-tocopherol and selenium [177]. However, in the Beta-Carotene and Retinol Efficacy Trial (CARET) evaluating the efficacy of vitamin A and β-carotene among smokers (25,000 IU of vitamin A + 30 mg of β-carotene daily) reported a 28% increased risk for lung cancer incidence in the treatment group after 4 years of follow-up as compared with the placebo group and an increase in risk for total mortality. This study was stopped earlier than planned [178]. Similar results were also shown in the Alpha-Tocopherol, Beta-Carotene Cancer Prevention (ATBC) Study, which reported a 16% excess in incident lung cancer among smokers in the β-carotene supplement group (20 mg daily) as compared with those in the placebo group for an average of five to eight years of follow-up [179]. In the Japan Public Health Centre-based prospective study, higher levels of dietary retinol intake were associated with an increased risk of lung cancer in men [180]. Accordingly, the 2018 Third Expert Report by the World Cancer Research Fund (WCRF) and the American Institute for Cancer Research (AICR) had reported that the daily intake of vitamin A and β-carotene could be associated with the decrease of some cancer risk but supplements of β-carotene are a recognized cause of lung cancer in current and former smoker [181]. One possible explanation for the failure of these studies to demonstrate that large β-carotene supplement has a protective role is that these patients were smokers, workers exposed to asbestos, ingested more alcohol or that carotenoids could exert a pro-oxidant effect on the lungs or reduce the absorption of other nutrients. In ferrets, the association of β-carotene supplementation and tobacco smoking leads to elevated carotene oxidation products with an increase in indicators of cell proliferation in lung [182,183]. Therefore, treatments should take into consideration the possible interactions of vitamin A and/or provitamin A with xenobiotics that may enhance the toxicity or side effects of retinoids.

These studies, however, do not indicate that the deficiency of these compounds, although subclinical, could not be related with an increased risk of suffering from lung cancer. The relevance of these results to people who have never smoked or to the effects of β-carotene or retinol from food or multivitamins is not known. In fact, the ATBC study showed that the subjects who at the beginning of the study had the highest concentrations of β-carotenes in plasma had the lowest cancer rate of all the participants in the study. The elevation of lung-cancer incidence or death as a result of β-carotene and/or vitamin A supplementation does not seem to appear in other large randomized trials. In a randomized, double-blind, placebo-controlled trial (Physician’s Health Study) of β-carotene (50 mg on alternate days), the supplementation to male physicians during a 12-year span produced neither benefits nor harm in terms of incidence of cancer, cardiovascular disease, or death from all cases [184]. It is worthy to mention that, in all human trials, synthetic beta-carotene (all-*trans-*β-carotene) was administrated, while the natural β-carotene contains several isomers (mainly all-*trans*, 9-*cis* and 13-*cis* β-carotene) and more research is needed to fully understand the beneficial mechanisms in β-carotenoid physiology [185].

More recently, RA has been evaluated as a single-agent treatment and combined with other chemotherapy in patients with non-small-cell lung carcinoma. However, these studies have not concluded a clear benefit with the use of retinoids in this lung carcinoma [63]; therefore, further research is needed to determine the effects of vitamin A/RA as a single agent or in combination with other treatments on lung and other types of cancer.

In this context, we have shown that vitamin A status modulates the expression of proliferative genes, p53, p21 WAF1/CIF1 and cyclin D1 and that the mechanism consists in a direct regulation of RA on c-Jun expression, a positive regulator of cell proliferation and G1–S phase progression. Our results indicated that vitamin A could regulate lung tumorigenesis and, moreover, this is in agreement with the significant role that retinoids play in cell proliferation and differentiation, emphasizing the importance of maintaining vitamin A tissue levels in a normal range [93].

### 4.6. Lung Fibrosis

Lung fibrosis is a major health problem worldwide, characterized by a replacement of normal lung parenchyma with fibrotic tissue accompanied by inflammation and excessive collagen deposition. It causes a progressive impairment of the pulmonary function leading to irreversible decrease in oxygen diffusion capacity of the lung, having it a poor prognosis with limited therapeutic options. This disease may be a secondary effect of other diseases or conditions, such as viral infections, sarcoidosis, environmental inhalants, radiation from cancer treatment, certain drugs, or it may appear without any known cause (idiopathic pulmonary fibrosis, IPF). The most prominent characteristic in the pathogenesis of lung fibrosis is persistent alveolitis, accumulation of myofibroblasts and the deposition of excessive amounts of ECM. Myofibroblasts, fibroblasts that express some features of muscle differentiation, are derived from resident mesenchymal cells, bone marrow progenitors (fibrocytes) and epithelial cells undergone epithelial-mesenchymal transition (EMT) [186,187,188]. One of the primary functions of the ECM is to maintain tissue integrity and homeostasis of multicellular organisms and its plays an important role in regulating alveolarization, tissue repair and remodelling in pulmonary tissue. Therefore, changes in the structure or composition of the ECM can induce alterations in cell and organ responses, leading to the development or progression of disease.

Retinoid signalling participates in the expression of ECM proteins including collagen, laminin, entactin, fibronectin, elastin and proteoglycans both directly, acting on their gene promoters and indirectly, modifying the expression of profibrotic factors and also affects the expression of cell membrane ECM receptors. Consequently, an altered retinoid signalling induces changes in ECM/BM ultrastructure which are associated to fibrogenic activation in different organs and deterioration of tissue parenchyma. This can contribute to the disorders induced by VAD in organs and tissues [123]. In this line, there is currently a considerable interest in the balance between increased ECM production and impaired ECM degradation in the context of fibrotic lung disease. In an experimental model of chronic VAD rats, we showed a thickening of the alveolar BM with an increase in the total amount of both type I and type IV collagens and a deposition of ectopic collagen fibrils in the BM [32]. The levels of α chains of collagen IV were also increased compared to that found in control lungs and this change was chain-specific, showing a significant increase in the content of α1 (IV), α3 (IV), α4 (IV) chains but not in α2 (IV) and α5 (IV) chains. In addition, the mRNA content for each α-chain varied in a similar way, indicating a regulation at the transcriptional level of the synthesis of α (IV) collagen chains. However, unlike what occurs with collagen IV, laminins decreased in VAD lungs [33]. Moreover, in VAD, specific matrix metalloproteinases 2 and 9 (MMP2 and MMP9), which play an important role in the degradation and remodelling of the BM, were decreased. Additionally, the levels of the two of their tissue inhibitors 1 (TIMP1) and 2 (TIMP2) did no change in chronic VAD. Consequently, the deficiency of vitamin A decreases BM degrading capacity and, therefore, can justify, at least in part, the BM alteration observed [33]. In agreement with these findings, it has been reported that lungs of VAD rats show scattered inflammation with increased collagen in areas of interstitial pneumonitis [28].

The mechanism through which VAD alters ECM is not clearly stablished. Among the different possibilities suggested, an alteration in the transforming growth factor-β1 (TGF-β1)/Smad3 signalling pathway has been considered to play a central role and is associated with lung fibrosis. TGF-β1 via Smad signalling pathway can upregulate the expression of several collagens and also via non-Smad signalling can activate the expression of other ECM molecules and its composition. Additionally, TGF-β1 is an inducer of EMT in alveolar epithelial cells, which has been suggested as an early event in the development of pulmonary fibrosis. Moreover, TGF-β1, via an integration of the Smad3 and STAT3 signalling pathways stimulates the connective tissue growth factor (CTGF), a central mediator of ECM production. In agreement, increased levels of TGF-β1 have been found in VAD tissues such as kidney, lung and aorta [32,123,186,188,189,190,191,192,193].

Most of the VAD-induced alterations of ECM are reversed by RA, suggesting a possibility for their therapeutical use in the treatment of fibrosis [32,95,152,166,194,195]. In fact, RA exhibits anti-proliferative, anti-inflammatory, anti-migratory and anti-fibrogenic activities and ameliorates bleomycin-induced lung fibrosis by downregulating the TGF-β1/Smad3 signalling pathway in rats. Consequently, inhibition of TGF-β1/Smad3 signalling with a variety of biologics, including neutralizing antibodies, short-interfering RNAs and antisense oligonucleotides has been suggested to ameliorate fibrosis [186,187,188]. However, RA signalling is complex, the possible mechanisms for its beneficial effect on fibrosis are diverse, intricate and controversial and more studies are needed to elucidate the precise effect of RA in fibrosis.

## 5. New Insights in Vitamin A Deficiency and Epithelial–Mesenchymal Transition

**Figure 2:**
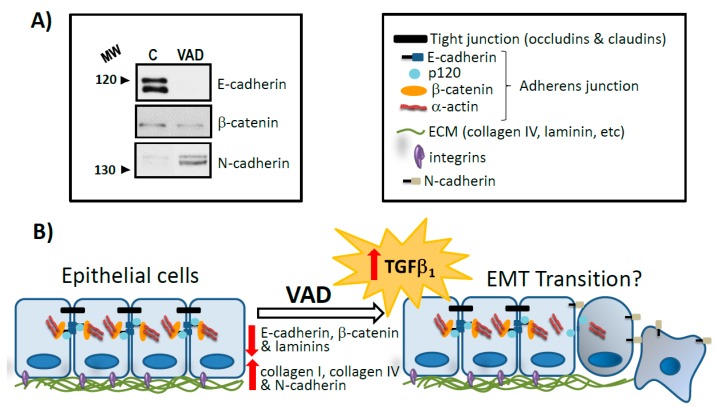
Retinoids are involved in epithelial-mesenchymal transition: (**A**) Markers of epithelial-mesenchymal transition (EMT) are observed in vitamin A-deficient (VAD) lungs. Lung protein extracts from control and chronic VAD rats were analysed by western blot to characterize EMT in this animal model. A decrease in E-cadherin and β-catenin protein levels was observed in VAD lungs, together with increased levels of N-cadherin, all of them hallmarks of EMT. A representative experiment is shown (*n* = 3). MW, molecular weight; (**B**) Epithelial-mesenchymal transition (EMT) in vitamin A-deficient (VAD) lung. VAD induces the activation of transforming growth factor β (TGF-β) which, in turn, drives the progression of the EMT observed. Basement membrane (BM) thickens and extracellular matrix (ECM) changes its composition in VAD lungs. Concomitant with these results, epithelial cells loss cell junctions and express mesenchymal markers, favouring the disassembly of the epithelial barrier and the migration of these newly formed mesenchymal cells.

EMT is the differentiation switch by which polarized epithelial cells differentiate into contractile and motile mesenchymal cells. EMT plays a key role during lung development and many diseases such as COPD, pulmonary fibrosis and lung cancer [196,197]. Historically, several stimuli from the local microenvironment, including growth factors and cytokines, inflammation, hypoxia, disruption of cell contact or contact with the surrounding ECM have been proposed as potential triggers of EMT. Although the molecular mechanisms underlying EMT in lung are still unclear, numerous studies have revealed that TGF-β signalling is a powerful inducer of EMT mostly through its canonical Smad-dependent pathway but also β-catenin signalling pathway seems to be involved [198,199]. This complex signalling network controls the expression of EMT markers. Hallmarks of EMT include the loss of expression or function of E-cadherin and reduced abundance of tight junction proteins and cytokeratins, as well as concomitant increase in abundance of mesenchymal markers, such as vimentin, fibronectin, α-smooth muscle actin (α-SMA) and N-cadherin, among others [200,201,202]. Cadherins are cell surface glycoproteins with important functions in cell-cell adhesion and tissue architecture maintenance. In fact, E-cadherin is a key molecule involved in the formation of cell-cell adhesion complexes called adherent junctions, playing a fundamental role in the regulation of the epithelial phenotype. Loss of cell contact is one of the early events in EMT that triggers a change in cytoskeletal composition and an arrangement that alters cell polarity to form spindle-shaped cells. These newly formed mesenchymal cells invade their basal ECM and migrate into underlying tissues (Figure 2).

It is well established the role that plays RA in the inhibition of EMT [203,204]. However, little is known about the effect of VAD in this process. As previously demonstrated, chronic VAD leads to activation of TGF-β signalling, increased oxidative stress and leucocyte infiltration in lung [32]. All these data unveil the possibility that VAD induces EMT in pulmonary tissue, since the increase of TGF-β1 results in alterations of the structures and composition of the ECM and BM. To address the plausible role of VAD in this process, we have analyzed several proteins involved in cell-junctions and whose deregulation promotes EMT. In VAD lungs, there is a decrease in E-cadherin and β-catenin concomitant with an increase in N-cadherin protein levels (Figure 2A). As described above, this pattern of expression is characteristic of the EMT (Figure 2B), further reinforcing the hypothesis that EMT is taking place in VAD deficient lung and is an early stage common to several respiratory pathologies already associated with vitamin deficiency.

## 6. Conclusions

This review provides a summary of current knowledge on the effects of a deficiency of vitamin A as a contributory factor on the development and progression of pulmonary disease along with the molecular mechanisms implicated. According to the World Health Organization, VAD constitutes, with protein malnutrition, the most common nutritional disorder in the world. VAD is a significant public health problem affecting particularly children and women during pregnancy in developing nations. A large body of evidence has shown that vitamin A is an important factor playing a direct part in the complex process of differentiation and maturation of pulmonary tissue. VAD has been associated with histopathological changes predisposing to severe lung dysfunction and respiratory diseases, which represent a major global health problem. Moreover, our results in lung VAD show that the pattern of expression is characteristic of the EMT, an early stage common to several respiratory pathologies. Additionally, based on our and others’ results provided in the literature, several pathological features of chronic lung disease could be prevented or even partially reversed by the use of vitamin A supplements and/or RA. There is sufficient evidence to consider the vitamin A supplementation programs for at-risk populations with potentially important implications for global health policy and, in particular, for the prevention and therapy of several pulmonary diseases, and retinoids as molecules with future therapeutic potential in the treatment of lung pathologies. Although definitive clinical benefits have not been observed in the treatment with retinoids in some pulmonary diseases, different studies have shown a protective effect of RA in several respiratory pathologies. Future studies are likely to open major venues for development of strategies for the use of retinoids as therapeutical targets.
